# Improving the Durability of Lime Finishing Mortars by Modifying Them with Silicic Acid Sol

**DOI:** 10.3390/ma15072360

**Published:** 2022-03-22

**Authors:** Valentina Loganina, Olga Davydova, Roman Fediuk, Mugahed Amran, Sergey Klyuev, Alexander Klyuev, Linar Sabitov, Karina Nabiullina

**Affiliations:** 1 Department of Quality Management and Technology of Construction Production, Penza State University of Architecture and Construction, 440028 Penza, Russia; loganin@mail.ru (V.L.); _oda@mail.ru (O.D.); 2 Polytechnic Institute, Far Eastern Federal University, 690950 Vladivostok, Russia; 3 Peter the Great St. Petersburg Polytechnic University, 195251 St. Petersburg, Russia; 4 Department of Civil Engineering, College of Engineering, Prince Sattam Bin Abdulaziz University, Alkharj 11942, Saudi Arabia; m.amran@psau.edu.sa; 5 Department of Civil Engineering, Faculty of Engineering and IT, Amran University, Amran 9677, Yemen; 6 Department of Theoretical Mechanics and Strength of Materials, Belgorod State Technological University Named after V.G. Shukhov, 308012 Belgorod, Russia; kluyevav@yandex.ru; 7 Kazan Federal University, 420008 Kazan, Russia; sabitov-kgasu@mail.ru (L.S.); karina.nabiullina@kpfu.ru (K.N.); 8 Kazan State Power Engineering University, 420066 Kazan, Russia

**Keywords:** durability, hardened properties, green composite, fresh properties, lime mortars, silicic acid sol

## Abstract

Lime materials are in great demand for the restoration of the walls of historical buildings. However, lime coatings have insufficient resistance during operation. The purpose of this work was the modification of lime mortars with silicic acid sol in order to obtain more durable crystalline materials for construction purposes. A technology has been developed for obtaining a silica-containing additive, which consists in passing a liquid glass solution with a density of 1.053 kg/m^3^ through a cationic column and obtaining a silicic acid sol with a pH of 3–4 and a charge of (−) 0.053 V. The regeneration time and the amount of sol have been determined. Regularities of change in the radius of particles of silicic acid sol depending on age are determined. It is established that at an early age (up to 5 days), the radius of sol particles can be determined in accordance with the Rayleigh equation, and at a later age, in accordance with the Heller equation. The results of the calculation show that at the age of 1–5 days, the radius of the sol particles is 17.1–17.9 nm, and then the particles become coarser and the particle radius is 131.2–143 nm at the age of 19 days. The work of adhesion of silicic acid sol to lime and the heat of wetting are estimated. It is shown that the work of adhesion of water to lime is 28.9 erg/cm^2^, and that of the sol is 32.8 erg/cm^2^. The amount of heat Q released when lime is wetted with SiO_2_ sol is 15.0 kJ/kg, and when lime is wetted with water, it is 10.6 kJ/kg.

## 1. Introduction

For the restoration of the walls of buildings, lime compositions are widely used. Lime finishing coatings have high vapor permeability, and have good compatibility with previously finished surfaces [1,2,3]. However, lime coatings have insufficient resistance during operation [4,5,6].

The lime compositions Holvi, Kalcemur, Silakra-lime, as well as Antik 1 and Antik 2 and others containing targeted additives, produced by various commercial firms, are widely used for the restoration of cultural heritage sites [7,8,9]. To increase the service life of lime coatings, finely ground fillers, additives for various purposes, etc., are introduced into the formulation of lime compositions. The use of active mineral (pozzolanic) additives increases the durability of lime coatings, however, grinding additives to a high degree of dispersion causes an increase in energy consumption [10,11,12]. To accelerate the hardening and increase the strength of lime composites, additives (sodium aluminate, sodium fluoride, potassium carbonate, calcium chloride, amorphous alumina, fine amorphous silica, etc.) are introduced into the formulation [13,14,15].

In [16,17,18], to accelerate the process of lime hardening, it is proposed to introduce additives based on natural zeolites into the formulation of lime compositions. In [19,20,21], it was proposed to use synthesized zeolites in composites based on mineral binders. The authors have identified patterns of structure formation of the lime composite in the presence of additives based on synthetic zeolite, which additionally consist in the formation of calcium-silicates, sodium hydrates and minerals of the zeolite group, an increase in the amount of chemically bound lime by 8.74%.

In [22,23,24], it was proposed to use synthesized calcium silicate hydrates (CSH) to increase the durability of lime coatings. A lime composition has been developed for finishing and restoring the walls of buildings in the form of a dry mixture containing a filler based on CSH and allowing to obtain mortar mixtures with a water-retaining capacity of 98–99%, drying time of 15–20 min, pot life 1–1.5 h. Coatings based on the proposed dry mortar (DM) are characterized by a vapor permeability coefficient of 0.05 mg/m·h·Pa, adhesion strength of 0.6–0.9 MPa, compressive strength of 3–4 MPa.

In China, during the restoration of historical buildings in the cities of Shanghai and Hangzhou, a system consisting of adhesives and injection mortars based on hydraulic lime (HL) was used to repair and restore exfoliated surfaces made of natural stone, gypsum and brick [25,26]. The authors set technical requirements for surface repair compositions: peel strength ≥ 0.1–0.5 MPa, ≤1.0 MPa (high peel strength can cause more cracking inside the stone surface), water absorption by capillary suction ≥2 kg/m^2^, vapor permeability μ ≤ 100, coefficient of thermal expansion—±50% of limestone.

The book [27] proposes lime compositions for restoration work, the formulation of which includes organic components (polysaccharides, proteins and fatty acids). The authors found that the addition of animal glue as an additive increases the mechanical strength of the mortar by 2 times, increases the carbonation front by 2 times, reduces porosity and reduces the pore size. To restore historical masonry, it is proposed in [28,29,30,31] to use lime-metakaolin mortars. Lime-metakaolin mixtures have been used to produce some of the Genoese white plasters. It has been established that as the ratio of metakaolin/lime in mortars increases, there is an increase in chemically bound water, a decrease in the pore size (less than 0.1 μm), and an increase in the compressive strength of mortars up to 9 MPa.

In recent literature on lime and carbonates used in cement materials, the papers of Songhui Liu and Yuli Wang stands out [32,33,34]. Paper [35] investigated the effects of calcium bicarbonate on the properties of ordinary Portland cement paste. Researchers [36] made comparison of Effects of Sodium Bicarbonate and Sodium Carbonate on the Hydration and Properties of Portland Cement Paste. Stefanidou et al. studied recycled sand in lime-based mortars [37]. Wang et al. [38] investigated effects of Aluminum Sulfate and Quicklime/Fluorgypsum Ratio on the Properties of Calcium Sulfoaluminate (CSA) Cement-Based Double Liquid Grouting Materials. Palomar et al. developed lime-cement mortars for coating with improved thermal and acoustic performance [39]. Aköz et al. researched effects of sodium sulfate concentration on the sulfate resistance of mortars with and without silica fume [40]. Fortes-Revilla et al. modelled a slaked lime–metakaolin mortar engineering characteristics in terms of process variables [26]. Gleize et al. microstructural investigated of a silica fume-cement-lime mortar [41].

At present, when solving technological problems, more and more attention is paid to colloidal dispersions based on silicon dioxide [42,43,44]. The main idea of using the sol-gel system as an additive in concrete, a mortar based on mineral binders is to use the structure of the sol to create a reinforcing additional structural element in the concrete mix and concrete [45,46,47]. An additional structural element, which is a silicon oxide nanoparticle, which over time, as a result of the reaction with Ca(OH)_2_, passes into calcium silicate hydrates, and contributes to a significant (up to 30%) reduction in the number of pores from a size of 1 nm and above (filling occurs in pores by sol particles and products of its interaction) [38,39,40,41,42,43,44].

In this regard, the aim of the work was the modification of lime mortars with silicic acid sol in order to obtain more durable crystalline materials for construction purposes. To achieve this aim, it was necessary to solve the following tasks:

- Development of technology for obtaining silica-containing additives;
- Establishing patterns of change in the radius of particles of sol of silicic acid depending on age;
- Proposal of a method for assessing the activity of the sol;
- Identification of effective stabilizers of sol of silicic acid;
- Establishing of regularities of hardening of lime compositions with the addition of sol;
- Evaluation of the work of adhesion of silicic acid sol with lime and the heat of wetting.

## 2. Materials and Methods

### 2.1. Materials

The following materials were used to prepare the finishing composition:

- Slaked lime (fluff) (Atmis-sakhar, Penza, Russia), activity 71–84 mg/g, true density 2230 kg/m^3^, bulk density 280 kg/m^3^, with specific surface 5590 cm^2^/g [48,49].
- For comparison, Aerosil 150 commercial pyrogenic colloidal silicon dioxide (Aerosil, Moscow, Russia) was used [50,51].
- When using the sol as an additive in lime compositions, a silicic acid sol with a pH of 4.5–5 with a density of 1013 kg/m^3^ was used [52,53].

The proportions of the compositions are given in Table 1.

### 2.2. Methods

**Figure 1:**
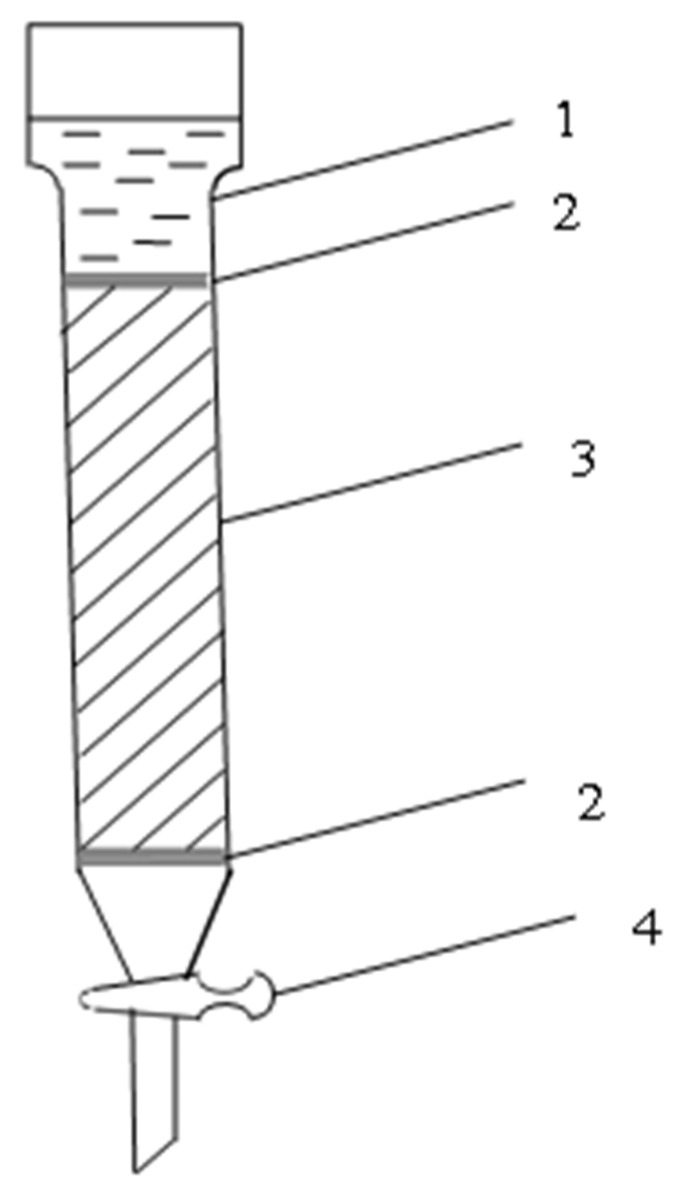
Ion exchange column: 1—sodium silicate solution; 2—glass wool; 3—cation resin; 4—crane.

Silicic acid sol was obtained using an ion-exchange column (Figure 1) filled with cation-exchange resin [45,54,55]. Before filling the column, the cation exchanger was treated as follows: it was placed in a beaker and filled with a 5% sodium hydroxide solution. After 3 h, the alkali was decanted and the cationite was thoroughly washed with water until the dark low molecular weight fractions were removed. After that, they were filled with a 10% hydrochloric acid solution and left to stand for a day until swelling.

A glass wool swab (2) was placed at the bottom of the column to retain the cation exchanger bed but allow the solution to flow freely. A cationite (3) was placed on glass wool, which was covered with glass wool (2) and glass beads to prevent it from swelling when the solution was poured. The liquid level in the column did not fall below the upper boundary of the cation exchanger.

The prepared column with H-cation exchanger was washed with distilled water until the acid reaction disappeared. A sodium silicate solution of a certain concentration was prepared from liquid glass with a density of 1.48 g/cm^3^. Then, the sodium silicate solution was placed in the upper part of the column and the valve was opened enough to obtain the required flow rate of the liquid through the ion exchanger.

Surface tension was determined by drop counting (stalagmometric method). From a special capillary-stalagmometer, the same volumes of water of the investigated liquid or solution are squeezed out. The number of drops formed from the same volume of liquid is proportional to the density of this liquid and inversely proportional to surface tension. Drops were counted five times and the arithmetic mean was calculated. The value of the surface tension of the investigated liquid was calculated by the formula:

(1) $$\sigma_{sol}=\sigma_{c}\frac{n_{c}}{n}$$

where $\sigma_{c}$—surface tension of the solvent, J/m^2^; $n_{c}$—the number of solvent drops in 1 mL; $n_{}$—the number of drops of the solution in 1 mL.

A distilled water with density $\rho_{m}^{20{}^{\circ}C}$ = 0.9982 g/cm^3^ and surface tension $\sigma^{20{}^{\circ}C}$ = 72.8 mN/m was used as reference liquid.

The wetting ability of the SiO_2_ sol was measured by the wetting angle (contact angle θ). The contact angle was determined microscopically. For this purpose, the diameter of the base of a drop of SiO_2_ sol on a marble substrate and its height were determined. The value of the contact angle θ was found by the formula (for $\theta <90^{0}$):

(2) $$tg\theta =\frac{2lh}{l^{2}-h^{2}}$$

where l—half the diameter of the base of the drop, m; and h—height of a drop (segment), m.

The electrokinetic potential (ζ-potential) was used in studies of the stability of silicic acid sol and was calculated based on the results of electrokinetic measurements obtained in the study of electrophoresis as the movement of charged particles in an electric field. ζ-potential was determined depending on the aging period of silica sol. The physical meaning of the ζ-potential is defined as the work that must be expended to transfer a unit charge from a point in the solution volume with a potential equal to zero to a slip plane with a potential equal to ζ.

(3) $$\xi =\frac{\eta hL}{\varepsilon_{0}\varepsilon V\tau },$$

where *η* is a viscosity of the medium, Pa·s; *h* is a path traveled by the sol boundary; *L* is a distance between the electrodes in the solution; *ε*_0_ is an electrical constant, F/m; *ε* is a relative permittivity of the medium; *V* is a potential difference between the electrodes, V; and *τ* is a time, s.

The sol coagulation threshold was determined by the turbidimetric method using a FEK-56M photoelectrocolorimeter (FEK, Moscow, Russia). The rapid coagulation threshold was found from the electrolyte volume *V_k_*, at which the optical density of the sol reaches its maximum value. The value of the coagulation threshold *c_k_* was calculated by the formula:

(4) $$c_{k}=\frac{c_{el}V_{k}}{V}$$

where *c_el_* is a concentration of the introduced electrolyte, mol/L; and *V* is a volume of the sol, mL.

A Dewar vessel was used to determine the heat of wetting. The amount of heat was calculated by the formula:

(5) $$Q=\frac{c\Delta tm_{H}}{m}$$

where *c* is a specific heat capacity, kJ/(kg °C); Δ*t* is a temperature change, °; *m_n_* is a sample mass, kg; and *m* is a mass of lime, kg.

## 3. Results and Discussion

Colloidal (SiO_2_)_n_ consists internally of [SiO_4_]^4−^ tetrahedra. On the surface, it is hydrated by the addition of H^+^ cation exchangers to unsaturated oxygen in the Si–O– ion to form OH. In a neutral medium, these particles are almost uncharged [19]. In an alkaline environment, the H^+^ cation is replaced by the Na^+^ cation. Due to the small charge, large ionic radius, and tendency to hydration, the Na^+^ cation passes into the diffuse layer of counterions, and the sol becomes negatively charged. The physical meaning of the electrokinetic, or zeta-(ζ-) potential is defined as the work that must be expended to transfer a unit charge from a point in the solution volume with a potential equal to zero to a slip plane with a potential equal to ζ. It has been established that the electrokinetic potential is (−) 0.053 V.

In this way, a technology has been developed for obtaining a silica-containing additive, which consists in passing a liquid glass solution with a density of 1.053 kg/m^3^ through a cation exchange column and obtaining a silica acid sol with a pH of 3–4 and a charge of (−) 0.053 V. The regeneration time and the amount of sol were determined.

**Figure 2:**
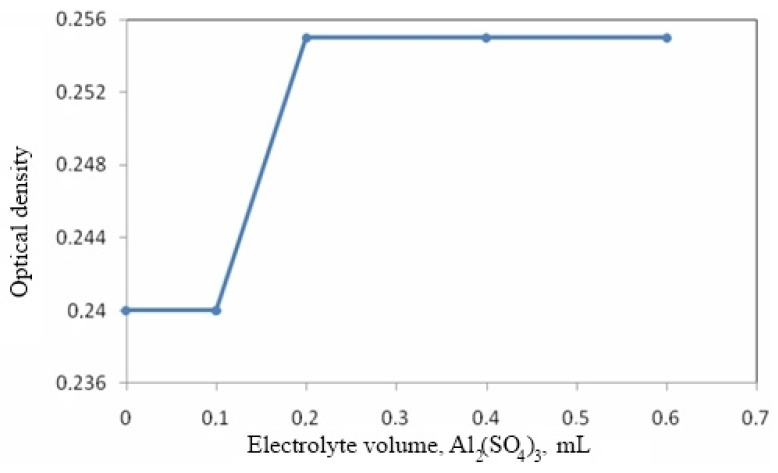
Dependence of optical density on the volume of electrolyte-coagulator.

The results of determining the coagulation threshold are shown in Figure 2.

It has been established that the amount of electrolyte Al_2_(SO_4_)_3_, which causes coagulation of the sol, is *c_k_* = 1.168 × 10^−6^ mol/L.

The assessment of the effect of stabilizers was carried out by the turbodimetric method. Effective stabilizers of silicic acid sol—gelatin and PVA—have been identified, which make it possible to prevent coagulation of silicic sol. The threshold of coagulation and the protective number were determined.

**Figure 3:**
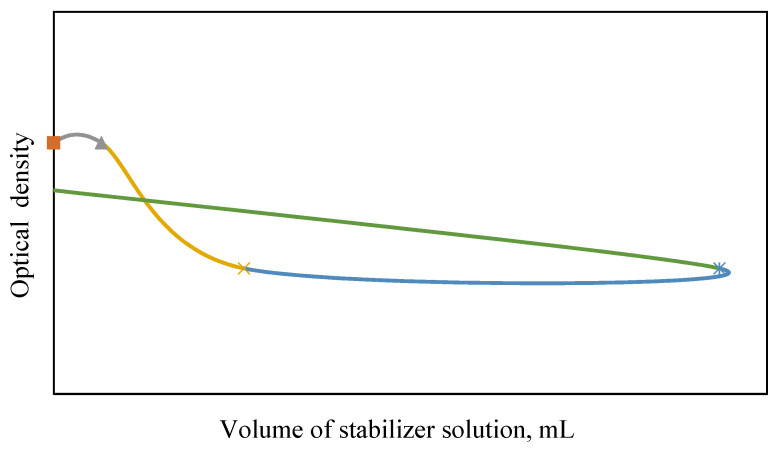
Dependence of optical density on the volume of PVA stabilizer.

**Figure 4:**
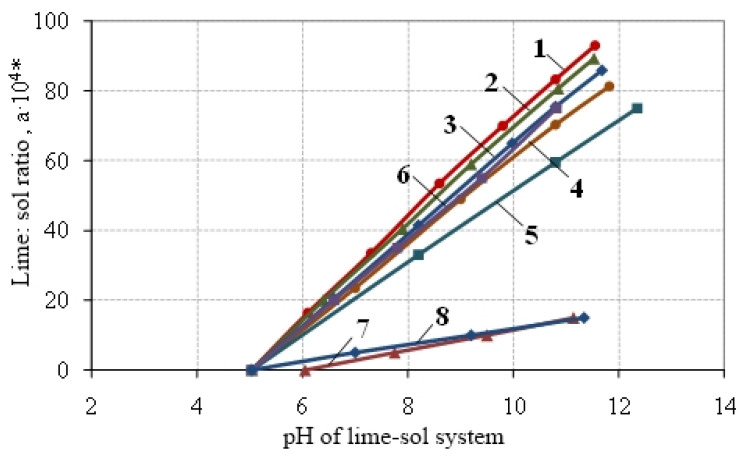
The kinetics of changes in the pH of the lime—sol system, depending on the ratio of lime: sol and the aging period of silica sol: 1—1 day; 2—5 days; 3—10 days; 4—15 days; 5—19 days;.6—stabilized sol; 7—Aerosil; 8—Aerosil heated. Note. * the ratio of the mass of lime to the mass of 2% silicic acid sol is given along the *y*-axis.

It has been established that gelatin and polyvinyl alcohol (PVA) are effective stabilizers for silica sol (Figure 3 and Figure 4). The protective ability of gelatin and PVA relative to the selected sol is characterized by the protective number *S* that is the amount of substance required to stabilize a unit volume of the sol. The protective number *S* (g/L sol) was calculated according to the equation [2]:

(6) $$S=\frac{c_{st}\cdot V_{prot}}{V}$$

where *c_st_* is a concentration of the stabilizer solution, g/L; and *V_prot_* is a volume of the stabilizer solution required to prevent coagulation of the sol, mL.

The results of calculation and experiment showed that the protective number of gelatin and PVA, S = 3.81 and 0.41 g/L of the sol, respectively.

Table 2 shows the values of the radius of the sol particles depending on the aging period.

Regularities of change in the radius of particles of silicic acid sol depending on age are determined. It has been established that at an early age (up to 5 days) the radius of sol particles can be determined in accordance with the Rayleigh equation, and at a later age, in accordance with the Heller equation. The results of the calculation show that at the age of 1–5 days, the radius of the sol particles is 17.1–17.9 nm, and then the particles become coarser and the particle radius is 131.2–143 nm at the age of 19 days.

A method for assessing the activity of the sol have been proposed, which consists in the potentiometric determination of the pH of the “sol-lime” system and the calculation of the amount of sol chemically bound to lime.

Determination of the activity of the sol was carried out according to the following method, which consisted in determining its amount that went into interaction with lime. To assess the amount of silicic acid sol that went into interaction with lime, the change in the pH of the “lime—sol” system was determined. For this purpose, a sample of Ca(OH)_2_ was placed into a flask containing a certain volume of sol (sol mass is 40 g) with a known concentration and pH, and the pH of the system was measured using a pH meter. The amount of sol m was determined in accordance with the stoichiometric equilibrium according to the equation of interaction between lime and sol. Figure 4 shows the kinetics of changes in the pH of the “lime—sol” system depending on the lime content.

When polybasic weak acids interact with strong bases, which include Ca (OH)_2_, the position of the first equivalence point can be determined by the formula:

(7) $${\mathrm{pH}}_{1}=\frac{pK_{1}+pK_{2}}{2},$$

where *K*_1_ is the dissociation constant of H_2_SiO_3_ for the first stage; *K*_2_ is the dissociation constant for the second stage.

Since *K*_1_ = 2.2·10^−10^ and *K*_2_ = 1.6·10^−12^, then the pH of the first equivalence point will be 10.73. The amount of sol used for interaction with lime was calculated at pH corresponding to the first equivalence point, i.e., equal to pH_1_ = 10.73. Then, two multiplied this value, because in the position of the first equivalence point, the amount of sol is ½ of its total amount that interacted with lime [7]. The calculation took into account the activity of lime. Table 3 shows the numerical values of the activity of the sol depending on its age.

Additionally, the activity of the silicic acid sol was evaluated by the change in the compressive strength of the samples of lime compositions with the addition of the sol, depending on its aging period. Samples were prepared with a ratio of components lime:sand = 1:3 and water:lime = 2 with the addition of 2% sol at a ratio of lime:sol = 1:0.25, lime:sol = 1:0.5 and lime:sol = 1:1. The samples were cured under air-dry conditions. The test results are given in Table 4.

It has been established that the use of an “older” sol leads to a decrease in compressive strength, so with a ratio of lime:sol = 1:1, the compressive strength at the age of 28 days when using the sol with an aging period of 1 day is 1.7 MPa, and when using a sol with an aging period of 15 days—1.1 MPa.

An additional confirmation of the dependence of the high activity of the sol is provided by the calculation data of the root-mean-square shift of the particle $\bar{\Delta }$. The root-mean-square shift of a particle over a time interval τ was determined according to the Einstein—Smoluchowski law:

(8) $${\bar{\Delta }}^{2}=2D\tau$$

where *D* is the diffusion coefficient; *τ* is time, s.

The diffusion coefficient *D* of a dispersed particle was calculated using the Einstein equation:

(9) $$D=\frac{kT}{6\pi \eta r}$$

where *k* is the Boltzmann constant, equal to 1.38 × 10^−23^ J/K; *T* is temperature, K; *η* is the viscosity of the medium, Pa s; and *r* is the particle radius, m.

At *r* = 72 nm,

(10) $$D=\frac{1.38\times 10^{-23}\times 293}{6\times 3.14\times 10^{-3}\times 72\times 10^{-9}}=2.98\cdot 10^{-12}\;m^{2}/s$$

(11) $$\bar{\Delta }=\sqrt{2\times 2.98\times 10^{-12}\times 10}=7.72\cdot 10^{-6}\;m$$

The calculation results showed that for 10 s the root-mean-square (RMS) shift of a particle with a radius of 72 nm was 7.72 × 10^−6^ m. The root-mean-square shift of a particle with a radius of 17 nm was 1.89 × 10 aging 1 day (Table 5).

When developing the formulation of lime compositions, the ratio of lime:sand components varied from 2 to 4. To study the patterns of formation of the structure and properties of finishing compositions and coatings based on them, compositions with different lime:sol (l:s) ratios were studied. For comparison, compositions with the addition of Aerosil were also investigated.

It has been established that according to the complex of rheological, technological and physical–mechanical properties, the ratio lime:sol = 1:1 is optimal. The introduction of a sol additive (the age of the sol is 1 h) helps to increase the compressive strength of lime mortars. The compressive strength of the composition with a ratio of components lime:sand = 1:3, water/lime = 2 with the addition of sol at a ratio of l:s = 1:0.5 at the age of 7 days was R = 0.68 MPa and at the age of 56 days R = 0.95 MPa, while in the control, respectively, R = 0.25 MPa and R = 0.61 MPa.

An increase in the age of the sol to 11 days reduces the effectiveness of the additive and causes a smaller increase in compressive strength at the age of 28 days of air-dry hardening, amounting to 39% at a ratio of l:s =1:1 (Table 6). The introduction of the Aerosil additive contributes to an increase in compressive strength at the age of 28 days of curing by only 9.8% compared to the control composition.

An increase in the strength of lime compositions with the addition of a silica sol, in our opinion, is due to the physicochemical interaction of the sol with lime. We determined the amount of free lime Ca(OH)_2_ in the hardened lime composition (Table 7). For this purpose, samples of a lime mortar with a composition of 1:4 were molded (control and with the addition of sol at a ratio of lime:sol = 1:1). After curing in air-dry conditions for 28 days, a sample of samples was ground and placed in a desiccator to exclude their further carbonation.

Regularities of hardening of lime compositions with the addition of sol have been established. The calculation results show that the content of free lime in the control compositions is 49.8% of the mass of lime used for kneading, and with the addition of sol is 39.6%, which confirms the chemical interaction of lime with sol.

In continuation of further studies to assess the physicochemical interaction of lime with silicic acid sol, the work of adhesion of the sol to lime and the heat of wetting were evaluated.

The work of the liquid to lime was calculated by the formula:

*A* = *σ* (1 − cos *θ*), (12)

It has been established that the contact angle of wetting the lime substrate with sol is 58°, and with water 53°, the surface tension of the sol practically does not differ from the value of the surface tension of water and is 69.8 erg/cm^2^. The calculation results show that the work of adhesion of water to lime is 28.9 erg/cm^2^, and that of the sol is 32.8 erg/cm^2^ (1 erg = 10^−7^ J), which leads to the best conditions for the interaction of the sol with lime.

The calculation results showed that the amount of specific heat Q released when lime was wetted with SiO_2_ sol was 15.0 kJ/kg, and when lime was wetted with water, it was 10.6 kJ/kg. Higher values of the heat of wetting, in our opinion, are due to the additional heat released due to the interaction of lime with sol.

Higher values of the heat of wetting, in our opinion, are due to the additional heat released due to the interaction of lime with the sol.

**Figure 5:**
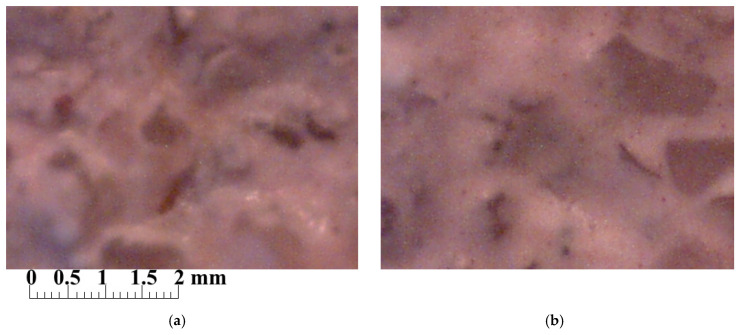
Photographs of the surface of coatings based on the control composition (**a**) and the composition with the addition of silicic acid sol (**b**), ×200.

When evaluating the structure of decorative plaster coatings, it was found that the control composition is characterized by greater porosity, and the pores are larger (0.25–0.43 mm). The composition with silicic acid sol has a more monolithic structure with pores 0.10–0.21 mm in size (Figure 5).

**Figure 6:**
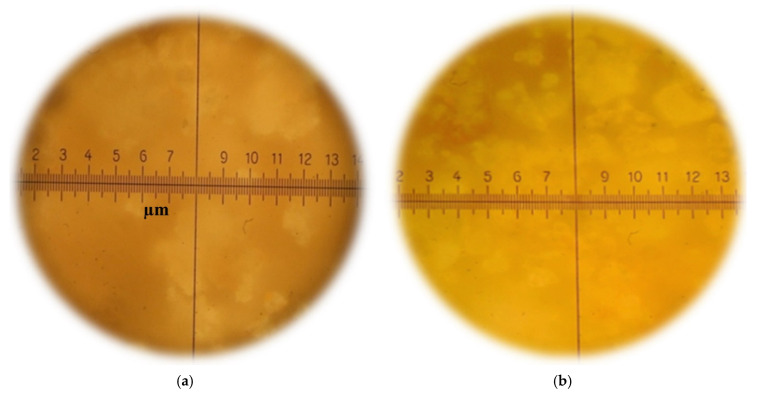
Micrographs of the fracture surface of coatings based on the control composition (**a**) and the composition with the addition of silicic acid sol (**b**), ×1000.

When evaluating the structure of coatings (Figure 6) based on the paint composition with the addition of silicic acid sol (b), the presence of areas characteristic of calcium silicate hydrates was established.

When analyzing the structure of coatings based on the control composition (a) and on the basis of the composition with the addition of silicic acid sol (b), Ca(OH)_2_ crystals are visible, and the crystals are larger in Figure 6a than in the photo of the coating structure with silica sol (Figure 6b). The color of the coating based on the composition with silicic acid sol becomes more saturated compared to the color of the coating based on the control composition.

## 4. Conclusions

Modification of lime finishing compositions with a complex additive based on silicic acid sol was carried out. The following main results were obtained:

1. A technology has been developed for obtaining a silica-containing additive, which consists in passing a liquid glass solution with a density of 1.053 kg/m^3^ through a cation exchange column and obtaining a silica acid sol with a pH of 3–4 and a charge of (−) 0.053 V. The regeneration time and the amount of sol were determined.
2. Regularities of change in the radius of particles of silicic acid sol depending on age are determined. It has been established that at an early age (up to 5 days), the radius of sol particles can be determined in accordance with the Rayleigh equation, and at a later age, in accordance with the Heller equation. The results of the calculation show that at the age of 1–5 days, the radius of the sol particles is 17.1–17.9 nm, and then the particles become coarser and the particle radius is 131.2–143 nm at the age of 19 days.
3. A method for assessing the activity of the sol has been proposed, which consists in the potentiometric determination of the pH of the “sol-lime” system and the calculation of the amount of sol chemically bound to lime.
4. Effective stabilizers of silicic acid sol—gelatin and PVA—have been identified, which make it possible to prevent coagulation of silicic sol. The threshold of coagulation and the protective number were determined.
5. Regularities of hardening of lime compositions with the addition of sol have been established. It has been revealed that the content of free lime in the control compositions is 49.8% of the mass of lime used for kneading, and with the sol addition of 39.6%, which confirms the chemical interaction of lime with the sol.
6. The work of adhesion of silicic acid sol to lime and the heat of wetting have been estimated. It has shown that the work of adhesion of water to lime is 28.9 erg/cm^2^, and that of the sol is 32.8 erg/cm^2^. The amount of heat Q released when lime is wetted with SiO_2_ sol is 15.0 kJ/kg, and when lime is wetted with water, it is 10.6 kJ/kg.

## Figures and Tables

**Table 1:** Mix proportions.

Lime:Sol		
1:0.25	1:0.5	1:1

**Table 2:** The values of the radius of the sol particles depending on the aging period.

Silica Sol Aging Time, Days	Radius of Sol Particles, nm
1	17
3	18
4	22
5	25
7	57
12	83
15	113

**Table 3:** Influence of silicic acid sol aging time on its activity.

Silica Sol Aging Time, Days	Sol Activity, %
1	83.3
5	80.7
10	75.5
15	70.3
19	59.8
Stabilized sol	78.1
Aerosil	10.7
Aerosil heated	9.8

**Table 4:** Values of compressive strength R, MPa.

Hardening Time, Days	Compressive Strength of Control Composition, mpa	Ratio Lime:Silicic Acid Sol					
		1:0.25		1:05		1:1	
		R	ΔR	R	ΔR	R	ΔR
7	0.25	0.33	0.08	0.45	0.20	0.54	0.29
		0.50	0.25	0.68	0.43	0.93	0.68
14	0.51	0.55	0.04	0.68	0.17	0.85	0.34
		0.72	0.21	0.97	0.46	1.25	0.74
28	0.85	0.88	0.03	0.93	0.08	1.10	0.25
		0.95	0.10	1.23	0.38	1.70	0.85

Note. Above the line are the values of compressive strength when using the sol with an aging period of 15 days, under the line—with an aging period of 1 day.

**Table 5:** RMS particle shift for sols of different ages.

Sol Aging, Days	Root-Mean-Square Particle Shift in 10 s, m
1	1.89 × 10^−5^
5	1.51 × 10^−5^
10	7.72 × 10^−6^
15	5.83 × 10^−6^
19	5.34 × 10^−6^

**Table 6:** Influence of additives on the compressive strength of lime compositions, MPa.

Aging Time, Days	Control Composition	Lime:Sol Ratio		The Amount of Aerosil in % of the Mass of Lime
		1:0.5	1:1	
7	0.25	0.35	0.43	0.26
14	0.31	0.44	0.56	0.36
28	0.51	0.57	0.71	0.6

**Table 7:** Results of determining the amount of free lime.

Composition	pH	OH- Ion Concentration, mol/L	The Content of Lime Ca(OH)_2_ in a Sample of 100 mg	The Content of Unbound (Free) Lime Ca(OH)_2_,%
Control	11.43	10^−2.57^	20 mg	49.8
Composition with sol	11.33	10^−2.67^	20 mg	39.6

## Data Availability

Data sharing not applicable.
